# The renal transport protein OATP4C1 mediates uptake of the uremic toxin asymmetric dimethylarginine (ADMA) and efflux of cardioprotective L-homoarginine

**DOI:** 10.1371/journal.pone.0213747

**Published:** 2019-03-13

**Authors:** Emir Taghikhani, Renke Maas, Martin F. Fromm, Jörg König

**Affiliations:** Institute of Experimental and Clinical Pharmacology and Toxicology, Friedrich-Alexander Universität Erlangen-Nürnberg, Erlangen, Germany; Medical University of Graz, AUSTRIA

## Abstract

Elevated plasma concentrations of the uremic toxin asymmetrical dimethylarginine (ADMA) and low plasma concentrations of L-homoarginine are independently associated with cardiovascular events and total mortality. Enzymes degrading ADMA [dimethylaminohydrolase 1 (DDAH1)] and synthesizing L-homoarginine [L-arginine:glycine amidinotransferase (AGAT)] are expressed in human proximal tubule cells. So far, it is not known which transport protein in the basolateral membrane of proximal tubule cells is mediating the uptake of ADMA into the cells for subsequent degradation or the export of intracellularly synthesized L-homoarginine. One study suggested that the uptake transporter OATP4C1 (gene symbol *SLCO4C1*) may be involved in the transport of ADMA and other uremic toxins. OATP4C1 is a member of the SLCO/SLC21 family of solute carriers, localized in the basolateral membrane of human proximal tubule cells. By using stably-transfected HEK cells overexpressing human OATP4C1, we demonstrate that ADMA and L-homoarginine are substrates of OATP4C1 with K_m_ values of 232.1 μM and 49.9 μM, respectively. ADMA and the structurally related uremic toxin SDMA (100 μM) inhibited OATP4C1-mediated L-homoarginine uptake (P < 0.01), whereas other tested uremic toxins such as urea and p-cresyl sulfate have no effect on OATP4C1-mediated transport. Preloading experiments (300 μM for 60 min) with subsequent efflux studies revealed that OATP4C1 also facilitates efflux e.g. of L-homoarginine. Both ADMA and L-homoarginine are substrates of human OATP4C1. Because proximal tubule cells are one site of ADMA metabolism and L-homoarginine synthesis, we postulate a protective role of OATP4C1 by mediating uptake of ADMA from and export of L-homoarginine into the systemic circulation.

## Introduction

L-homoarginine is an endogenous, non-proteinogenic amino acid, which may increase nitric oxide availability and enhance endothelial function [1]. In recent years, low urinary and circulation L-homoarginine concentrations emerged as a risk marker for renal and cardiovascular diseases and mortality [2,3]. L-homoarginine is endogenously formed from lysine and occurs in various body fluids and organs [4]. As demonstrated by Genome Wide Association studies (GWAS studies), one single-nucleotid polymorphism (SNP) in the gene encoding for the L-arginine:glycine amidinotransferase (AGAT) is associated with circulating L-homoarginine concentrations [5,6], indicating that AGAT is one major enzyme for endogenous L-homoarginine synthesis. The kidney is one important site of AGAT expression [7] and L-homoarginine plasma concentration is closely linked to glomerular filtration rate. Lower L-homoarginine concentrations were found in patients with impaired kidney function [8], suggesting that less L-homoarginine is synthesized in proximal tubule cells and exported into plasma in patients with reduced kidney function thereby indicating an important role of the kidney in L-homoarginine metabolism and homeostasis [2].

While lower L-homoarginine concentrations are independently associated with cardiovascular and all-cause mortality, high plasma concentrations of the structurally related arginine derivative and uremic toxin asymmetric dimethylarginine (ADMA) could be detected in CKD (chronic kidney disease) patients [9]. Since then, numerous studies have linked elevated ADMA plasma concentrations to cardiovascular events and total mortality [10]. ADMA is considered an important uremic toxin and its plasma concentration increases up to three-fold in advanced CKD [11]. Interestingly, the kidney is not only involved in the filtration but also in the metabolism of ADMA by dimethylaminohydrolase 1 (DDAH1) and alanine:glyoxylate aminotransferase 2 [AGXT2 [12]]. DDAH1 is expressed in human renal proximal tubule cells and likely responsible for the degradation of ADMA after it is taken up from blood into proximal tubule cells.

The uptake transporter OATP4C1 (gene symbol *SLCO4C1*) is the only member of the SLC21/SLCO family of transport proteins (SLC = solute carrier). An immunohistochemical analysis of kidney biopsies obtained from transgenic rats expressing human OATP4C1 showed, that OATP4C1 is located in the basolateral membrane of the proximal tubule epithelium of the S2 segment.

Using this transgenic rat model, an involvement of this transporter in the elimination of uremic toxins was studied [13]. The authors found, that the overexpression of human OATP4C1 in rat kidney reduced hypertension, inflammation and cardiomegaly after five-sixths nephrectomy, an established model of renal failure. Furthermore, this overexpression decreased the plasma concentrations of the uremic toxins ADMA, guanidine succinate, and trans-aconitate suggesting that several uremic toxins might be substrates of this transport protein. Finally, the authors concluded that the upregulation of OATP4C1 in patients with CKD might have a therapeutic potential by facilitating the renal excretion of uremic toxins that are substrates of this transporter [13].

Based on these findings and because OATP4C1-mediated transport of ADMA or L-homoarginine has not been studied so far, we established HEK cells stably overexpressing human OATP4C1. Using labeled ADMA and L-homoarginine as well as L-arginine, we investigated OATP4C1-mediated transport of these substances to gain more insights into the role of this transport protein for the homeostasis of ADMA and L-homoarginine, which are both acting antagonistically in the cardiovascular system.

## Materials and methods

### Materials

[^3^H]labeled ADMA (25 Ci/mmol) was from BIOTREND Chemikalien GmbH (Cologne, Germany), [^3^H]labeled L-arginine (43 Ci/mmol) and [^3^H]labeled digoxin (20 Ci/mmol) was purchased from American Radiolabeled Chemicals, Inc. (St. Louis, U.S.A.). [^3^H]labeled L-homoarginine (6 Ci/mmol) was from ViTrax (St. Jefferson, U.S.A.). Unlabeled ADMA, SDMA and L-arginine were from Enzo Life Sciences GmbH (Lörrach, Germany) and unlabeled L-homoarginine was obtained from Acros Organics (New Jersey, U.S.A.). Urea was purchased from Carl Roth GmbH & Co. KG (Karlsruhe, Germany), sodium butyrate was obtained from Merck KGaA (Darmstadt, Germany) and p-cresyl sulfate was provided by Prof. Dr. Roos Masereeuw from the Utrecht Institute for Pharmaceutical Sciences (Utrecht, The Netherlands). Dulbecco`s phosphate buffered saline, fetal bovine serum, geneticin-disulfate (G418), minimum essential medium, penicillin streptomycin solution and 0.05%-trypsin-EDTA (0.02%) solution were from Life Technologies GmbH (Darmstadt, Germany). All other substances were purchased from Sigma-Aldrich (St. Louis, USA) with the highest grade available. 12-well cell culture plates were from Greiner Bio-One (Frickenhausen, Germany). BCA Pierce Protein Assay Kit was obtained from Life Technologies GmbH.

### Antibodies

The polyclonal rabbit anti-human OATP4C1 antiserum AVV was directed against the carboxyterminal end of the OATP4C1 protein and was obtained from the Division of Tumor Biochemistry of the German Cancer Research Center (Heidelberg, Germany).

### Cell culture

HEK293 cells were cultured in minimal essential medium, which was supplemented with 10% heat-inactivated fetal bovine serum, 100 U/ml penicillin, and 100 μg/ml streptomycin. G418 (800 μg/ml) was used as selection antibiotic. Cells were incubated at 37°C and 5% CO_2_. The cells were routinely subcultured using trypsin 0.05%-EDTA (0.02%) solution.

### Generation of a HEK293 cell line stably overexpressing human OATP4C1

Cloning of the *SLCO4C1* cDNA encoding human OATP4C1 was performed using an RT-PCR approach with the primers oOATP4C1-RT.for (5`-CTCCTATAACTGTGTCTATCC-3`) and oOATP4C1-RT.rev (5`-CCCATTTCACCCTTCTTTTACT-3`). The obtained cDNA was cloned into the expression plasmid pcDNA3.1(+), sequenced and base pair exchanges leading to amino acid exchanges, compared to the reference sequence (accession number NM_180991.4) were corrected using the QuikChange Lightning Multi-site-directed mutagenesis kit (Agilent technologies, La Jolla, USA). The resulting plasmid pOATP4C1.31 contains the *SLCO4C1* cDNA encoding a human OATP4C1 protein 100% identical to the one encoded by the reference sequence. HEK293 cells were transfected with the Effectene^TM^ Transfection Reagent Kit (QIAGEN GmbH, Hilden, Germany) according to the manufacturer`s instructions. After selection with G418 (800 μg/ml) for 4 weeks, transfected HEK cells were screened for *SLCO4C1* mRNA expression using LightCycler-based quantitative RT-PCR as described [14]. These cells were used in control experiments to exclude cell specific effects.

### Immunofluorescence microscopy

Localization of the OATP4C1 protein was analyzed by confocal laser scanning microscopy and performed with an Axiovert 100 M microscope (Carl Zeiss GmbH, Jena, Germany) and the Zeiss LSM Image Browser version 4.2.0.121. HEK-OATP4C1 and HEK-VC cells were grown on poly-D-lysine coated object slides in cell culture plates at an initial cell density of 5 x 10^6^ cells/slide. After 24 h of incubation at 37°C, the cells were induced with 10 mM sodium butyrate [15]. Object slides were incubated with polyclonal rabbit anti-human OATP4C1 antiserum AVV (1:500 in H_2_O containing 2% bovine serum albumin) over night. Slides were subsequently incubated with a Cy3-conjugated goat anti-rabbit IgG FC secondary antibody (1:500 in H_2_O only, Dianova GmbH, Hamburg, Germany). The nuclei were counterstained with DAPI (100 ng/ml solution in H_2_O, AppliChem GmbH, Darmstadt, Germany).

### Immunoblot analysis

Immunoblot analysis was conducted as described before [14]. Human kidney tissue was used as positive control. Protein content of the obtained samples and the positive control was determined by using the BCA Assay according to the manufacturer`s instructions. After electroblotting, the membrane was incubated in 1:1 000 diluted polyclonal rabbit anti-human OATP4C1 AVV antiserum. Goat anti-rabbit IgG (1:10 000), conjugated with horseradish peroxidase (GE Healthcare Life Sciences, Buckinghamshire, UK), was used as secondary antibody. To detect β-actin, the membrane was incubated in stripping buffer (Thermo Scientific, Rockford, USA) and incubated in a 1:10 000 diluted monoclonal mouse anti-human β-actin primary antibody (Sigma-Aldrich, St. Louis, USA). Horseradish peroxidase-labeled goat anti-mouse IgG (Dianova GmbH, Hamburg, Germany) was used as secondary antibody in a dilution of 1:2 000. For protein detection, Clarity^TM^ ECL Western Blotting Substrate (Bio-Rad Laboratories Inc., Hercules, USA) was used. Digital quantification was conducted with ChemiDoc XRS detection system (Bio-Rad Laboratories GmbH).

### Transport assays & inhibition studies

HEK-OATP4C1 and HEK-VC cells were seeded into poly-D-lysine coated 12-well plates with an initial density between 5 x 10^5^ cells/well and 7 x 10^5^ cells/well, incubated for 24 h at 37°C and 5% CO_2_ and subsequently induced with 10 mM sodium butyrate [15]. Transport assays were performed as described earlier [16,17]. The measured substrate concentration in the lysat was normalized to the protein content, which was determined using BCA Assay.

During characterization of stably-transfected cells, digoxin was used in a concentration of 5 μM. Initial transport experiments using ADMA, L-arginine and L-homoarginine as potential substrates of OATP4C1 were performed with low (1 μM) and high (50 μM) substrate concentrations. Time dependency experiments were performed with incubation periods of 1, 2, 5 and 10 minutes. For the determination of the kinetic parameters, substrates were applied in concentrations between 1 μM and 500 μM with an incubation time of 2 minutes. To measure the potential influence of uremic toxins or of digoxin on the OATP4C1-mediated uptake, L-homoarginine was used as substrate (20 μM) and the tested uremic toxins were simultaneously applied in a concentration of 50 μM. Digoxin was used in a concentration of 5 μM. In additional studies the dose-dependent inhibitory effect of ADMA and SDMA on the OATP4C1-mediated uptake of physiological L-homoarginine concentrations was examined. Here cells were incubated with 2 μM of L-homoarginine and increasing concentrations of either ADMA or SDMA between 0.5 μM and 1000 μM. Based on these data IC_50_ values were calculated.

### Efflux transport assay

The OATP4C1-mediated efflux of ADMA, L-arginine and L-homoarginine was studied by preloading the cells with 300 μM of the compounds for 60 minutes. Cells were then washed with 1 ml of fresh prewarmed uptake buffer (37°C). Immediately after washing the cells, 800 μl of prewarmed uptake buffer were applied onto the cells for incubation periods of 2, 5 and 10 minutes. The transport activity was stopped by putting the cells on ice. The supernatant was collected and the cells were washed three times with cold uptake buffer and lysed. The radioactivity was measured in the lysate and in the supernatant by liquid scintillation counting. To verify that the observed efflux was caused by transporter activity and not by passive diffusion, the efflux of L-homoarginine was also investigated at an incubation temperature of 4°C.

### Statistical analysis

All data were expressed as means ± SEM. Unless stated otherwise, an unpaired, two-tailed *t*-test was conducted to analyze the statistical significance in the differences of cellular accumulation, mRNA expression and protein expression. Statistical significance was considered to be given at p-value < 0.05. Net uptake was calculated by subtracting the accumulation of the respective compound into the HEK-VC cell line from the accumulation into the HEK-OATP4C1 cell line. The percentagewise inhibition of OATP4C1-mediated transport caused by uremic toxins was calculated by comparing the inhibited transport to a control experiment, which was conducted in the absence of the respective inhibitor.

K_m_ and IC_50_ values were calculated by using GraphPad Prism (Version 5.01, 2007, GraphPad Software, San Diego, CA, USA).

The efflux data for every time point were calculated in relation to the original substrate content after preloading. This calculation has been done individually for the HEK-OATP4C1 cells and the respective control cell line.

The percentagewise content of the applied compound in the HEK-OATP4C1 and HEK-VC cells was plotted against the incubation time.

The amount of compound in the supernatant was analogously expressed for every time point in relation to the original content of the compound in the lysate.

## Results

### HEK293 cells recombinantly overexpressing human OATP4C1

**Fig 1:**
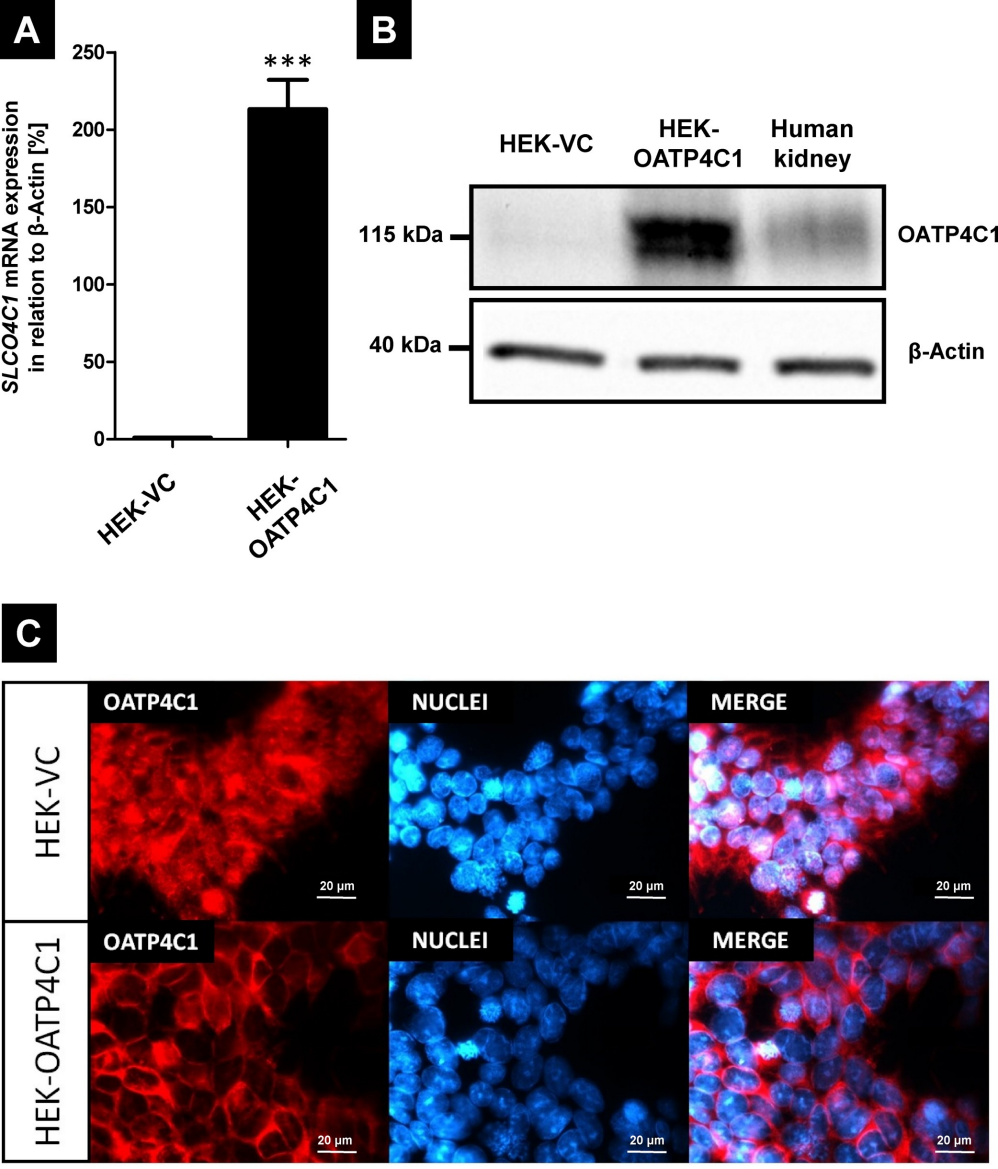
Characterization of HEK293 cells stably expressing the human renal transport protein OATP4C1 (gene symbol *SLCO4C1*). (A) Analysis of *SLCO4C1* (encoding human OATP4C1) mRNA expression. The expression is quantified relative to the expression of the housekeeping gene β-actin. HEK-VC = cell line transfected with the empty vector and selected under the same conditions serving as control cell line, HEK-OATP4C1 = cell line recombinantly overexpressing human OATP4C1. Data are expressed as means ± SEM. The experiment has been performed once with n = 3. ***P < 0.001. (B) Immunoblot analysis of OATP4C1 expression in the control cell line (HEK-VC), the cell line expressing human OATP4C1 (HEK-OATP4C1) and in a human kidney sample. (C) Confocal laser scanning microscopy analyzing the localization of OATP4C1 in the control cell line (HEK-VC) and in the transfectant (HEK-OATP4C1).

We first established HEK293 cells stably overexpressing human OATP4C1. After stable transfection and clone selection, the cell clone with the highest *SLCO4C1* mRNA (encoding OATP4C1) expression showed 213 ± 19% *SLCO4C1* mRNA expression relative to the expression of the housekeeping gene β-actin (Fig 1A). Immunoblot analysis demonstrated that this clone showed a strong overexpression of the OATP4C1 protein with a molecular mass in the same range as the endogenously synthesized OATP4C1 protein in human kidney samples (Fig 1B). The localization of the recombinantly expressed protein was analyzed by confocal laser scanning microscopy demonstrating a distinct membrane staining compared to the unspecific staining of the HEK-VC cells (Fig 1C).

**Fig 2:**
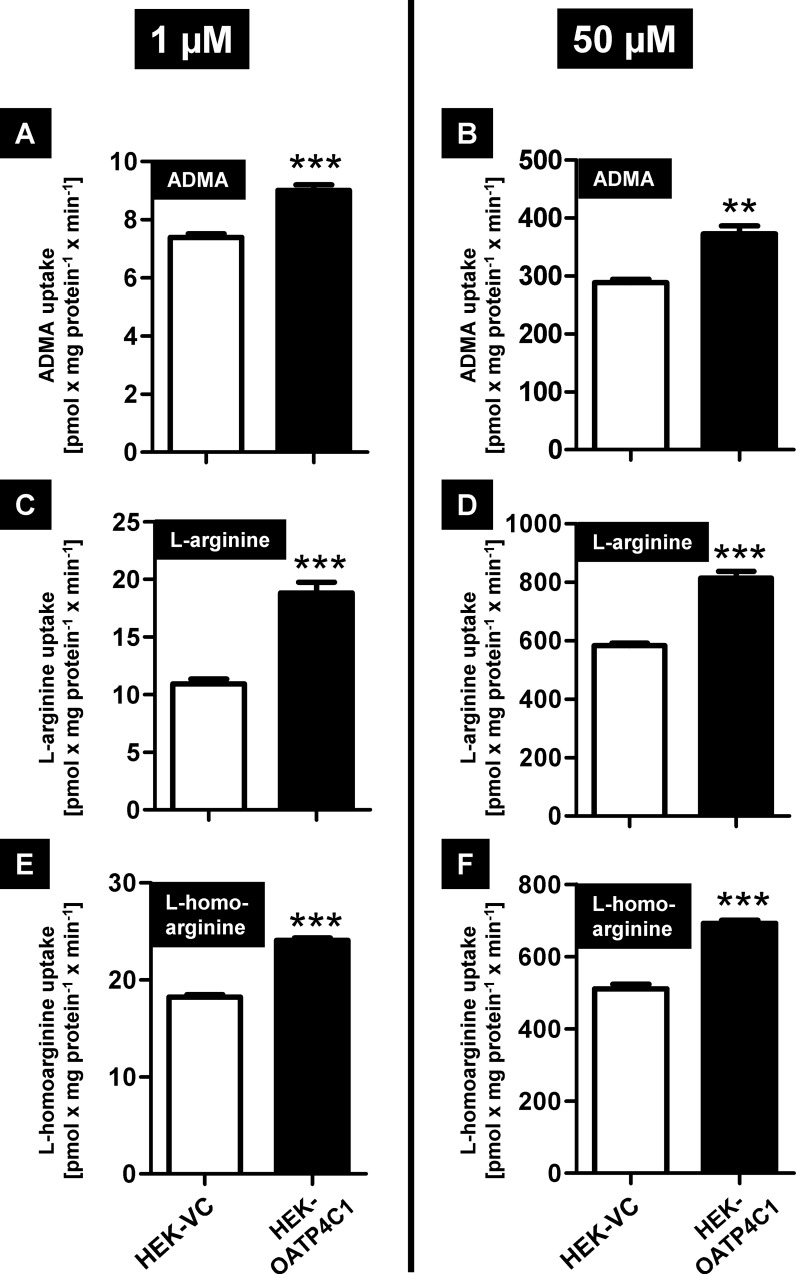
**Uptake experiments using 1** μ**M (A, C, E) and 50** μ**M (B, D, F) of the respective substance.** Black bars represent the uptake into the OATP4C1-expressing cell line (HEK-OATP4C1) and white bars represent the uptake into the control cell line (HEK-VC). Data are expressed as means ± SEM. Studies were performed in two experiments with n = 2 (n = 4). **P < 0.01; ***P < 0.001.

In uptake experiments, we observed a significantly higher accumulation of ADMA, L-arginine and L-homoarginine in HEK-OATP4C1 cells compared to HEK-VC cells at a low substrate concentration of 1 μM (Fig 2A, 2C and 2E), which is in the physiological range for ADMA and L-homoarginine and at a high concentration of 50 μM (Fig 2B, 2D and 2F).

### OATP4C1-mediated uptake of ADMA, L-arginine and L-homoarginine

**Fig 3:**
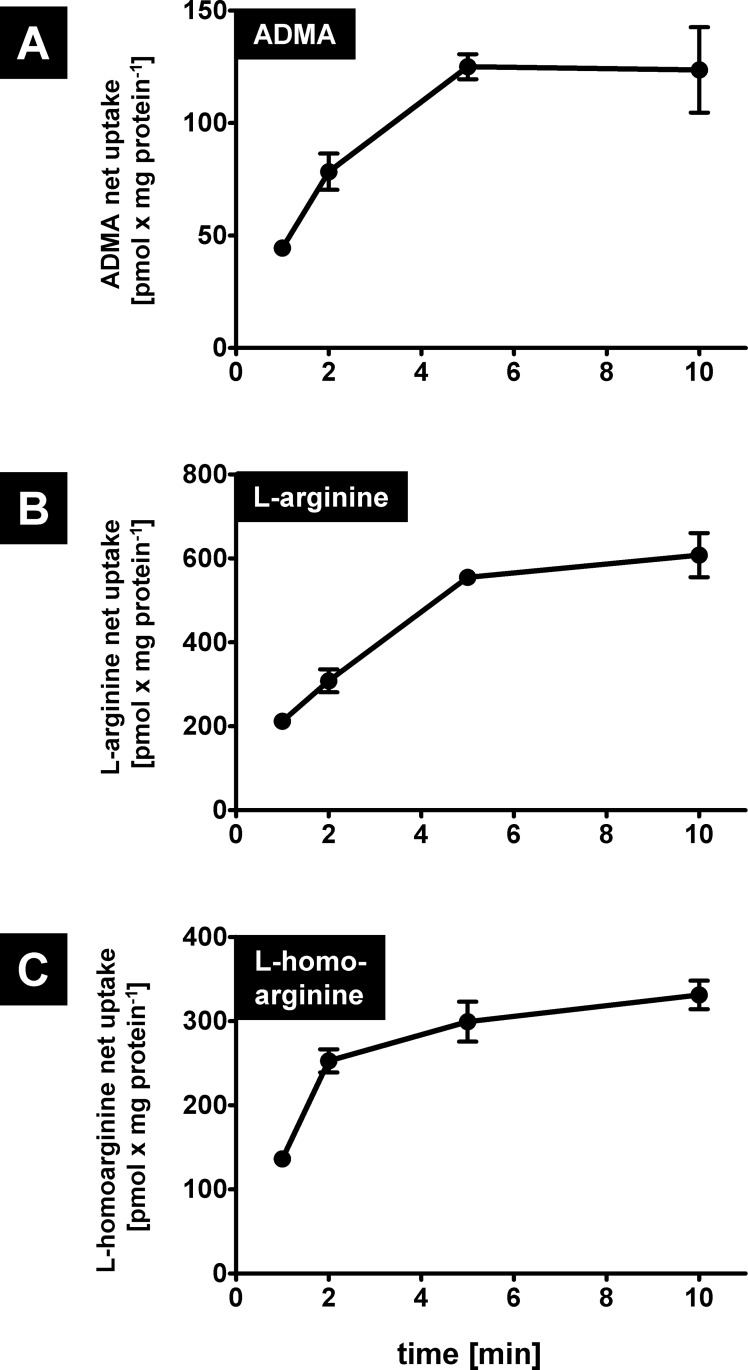
**Time dependency experiments for OATP4C1-mediated ADMA (A), L-arginine (B) and L-homoarginine (C) net uptake obtained from experiments with HEK-OATP4C1 and HEK-VC cells.** Shown are the net uptake values obtained at 1, 2, 5 and 10 minutes of incubation. Data are expressed as means ± SEM. Experiments were performed on two separate days with n = 3 each (n = 6).

Next, we investigated the time dependency of the OATP4C1-mediated uptake using a substrate concentration of 25 μM at incubation time points of 1, 2, 5 and 10 minutes. These uptake experiments demonstrated linearity of net uptake for ADMA and L-homoarginine (Fig 3A and 3C) up to two minutes and for L-arginine (Fig 3B) up to five minutes. Therefore, concentration dependency and inhibition experiments were conducted with an incubation time of two minutes.

**Fig 4:**
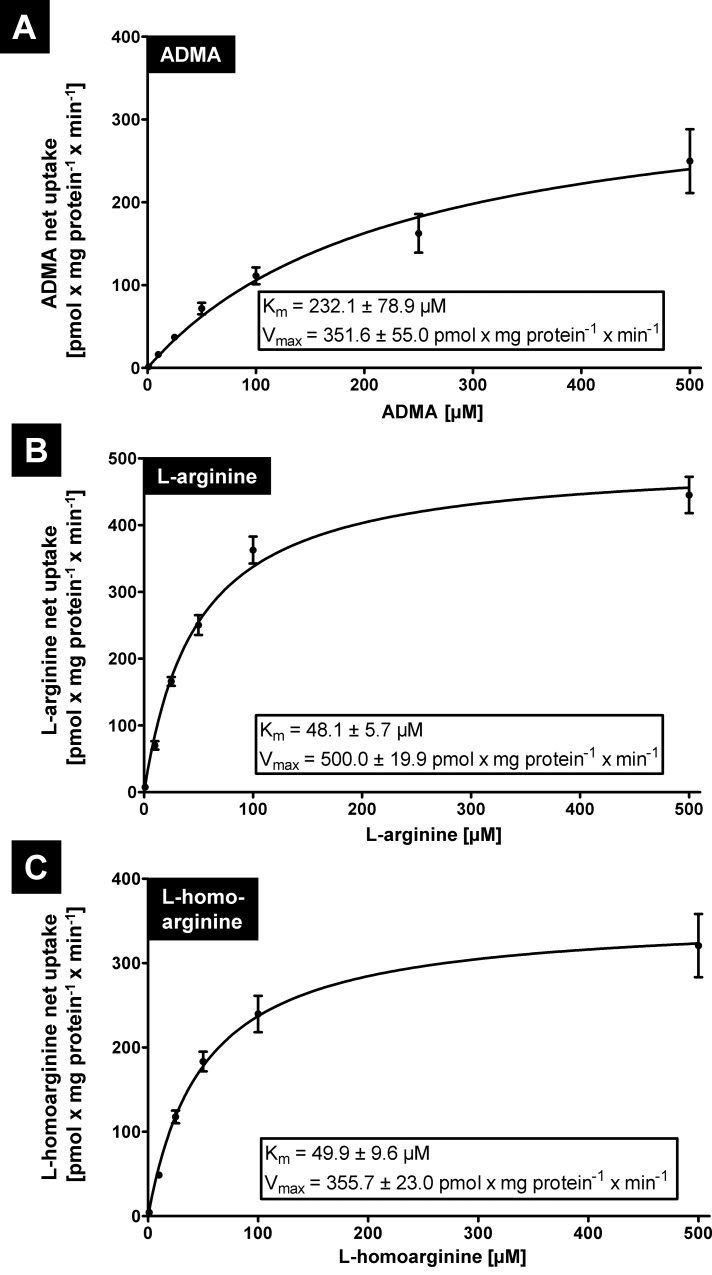
**Determination of kinetic constants (K**_**m**_ **values) for OATP4C1-mediated net uptake of ADMA (A), L-arginine (B) and L-homoarginine (C) obtained from experiments with HEK-OATP4C1 and HEK-VC cells.** Presented are net uptake values at indicated substrate concentrations. Data are expressed as means ± SEM. All experiments were performed on two separate days with n = 2 x 2 each (n = 8).

To determine kinetic parameters, concentration dependency studies were performed using substrate concentrations between 1 μM and 500 μM. For ADMA a K_m_ value of 232.1 ± 78.9 μM, for L-arginine a K_m_ value of 48.1 ± 5.7 μM and for L-homoarginine a K_m_ value of 49.9 ± 9.6 μM was calculated (Fig 4).

### Inhibition of OATP4C1-mediated L-homoarginine uptake by uremic toxins

**Fig 5:**
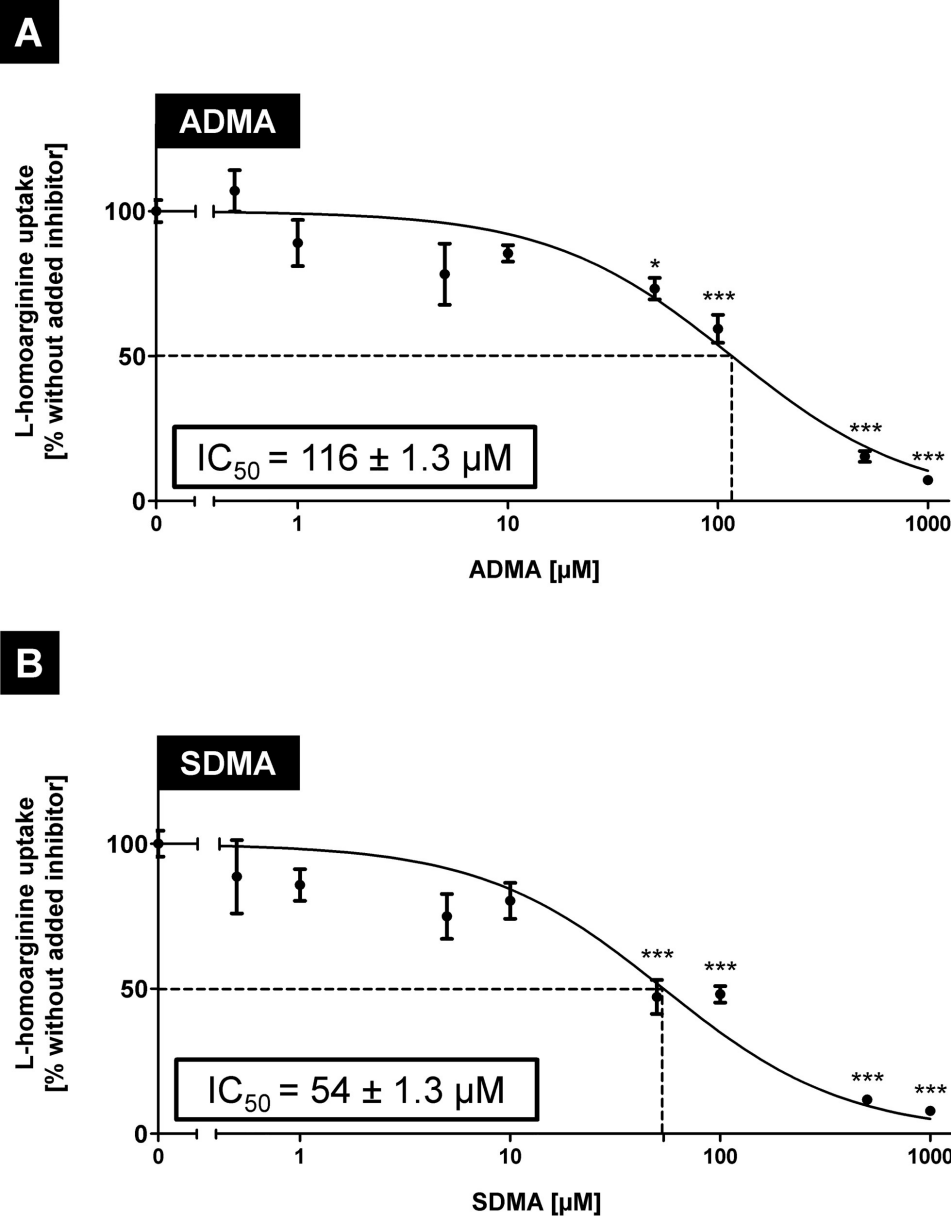
**Concentration-dependent inhibition of OATP4C1-mediated L-homoarginine (2** μ**M) net uptake by uremic toxins ADMA (A) and SDMA (B).** The calculated net uptake values were used to determine IC_50_ values. Data are expressed as means ± SEM. Experiments were performed on two separate days with n = 3 (n = 6). *P < 0.05; ***P < 0.0001; one-way ANOVA Dunnett`s multiple comparison test.

Next, we investigated if the OATP4C1-mediated net uptake of L-homoarginine (2 μM) could be inhibited by ADMA and the related uremic toxin SDMA. Both substances were applied in concentrations between 0.5 and 1000 μM and IC_50_ values were determined (Fig 5A and 5B). ADMA and SDMA inhibited OATP4C1-mediated L-homoarginine uptake in a concentration-dependent manner with IC_50_ values of 117 ± 1.3 μM and 54 ± 1.3 μM for ADMA and SDMA, respectively. Significant inhibition was observed at a concentration of 50 μM ADMA and SDMA but even at lower ADMA and SDMA concentrations inhibition of OATP4C1-mediated L-homoarginine uptake could be detected.

**Fig 6:**
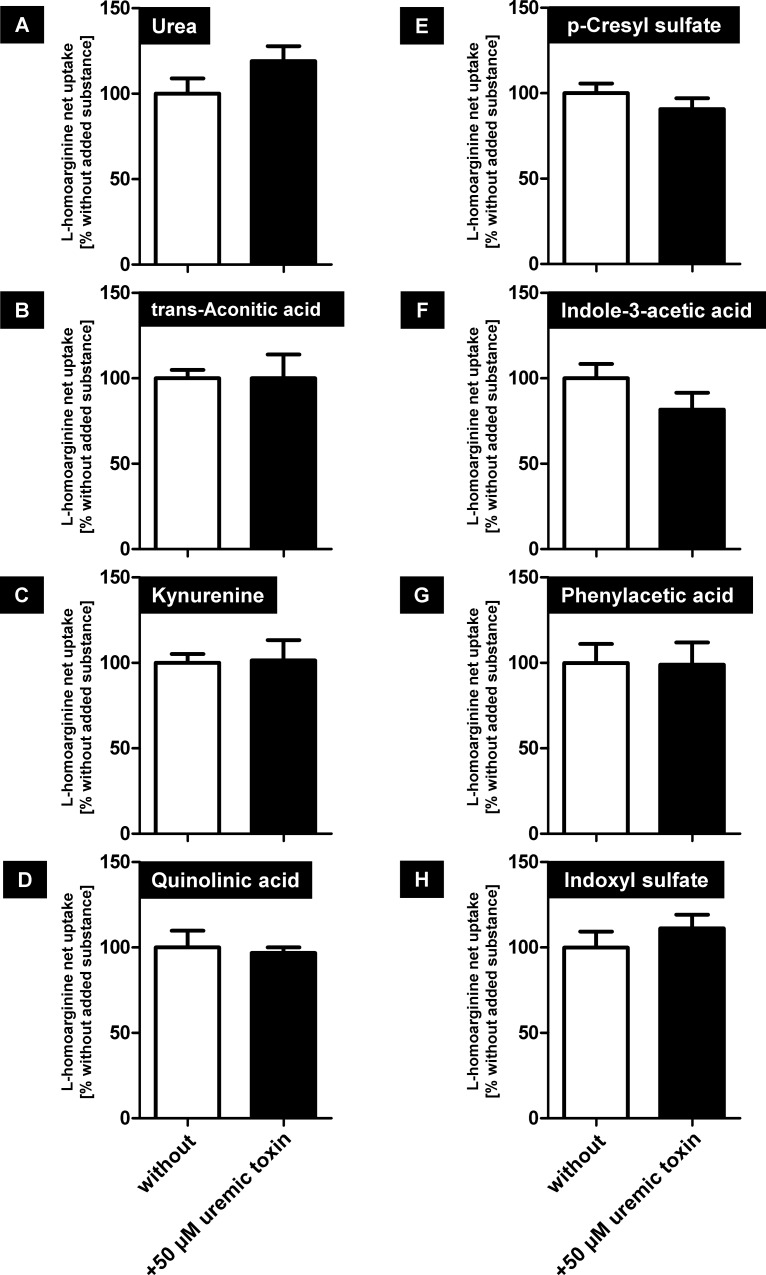
Analysis of a possible interaction of uremic toxins with OATP4C1-mediated L-homoarginine net uptake. All toxins were added in a concentration of 50 μM. Black bars represent the uptake with added uremic toxin (+ 50 μM uremic toxin) and white bars represent uptake without added putative inhibitor (without), determined under the same experimental conditions. Data are expressed as means ± SEM. All experiments were performed in two experiments with n = 2 (n = 4).

Several other uremic toxins such as kynurenine and indoxyl sulfate showed no significant modulation of OATP4C1-mediated L-homoarginine net uptake (Fig 6A–6H) at the tested concentration of 50 μM.

### OATP4C1-mediated efflux of ADMA and L-homoarginine

**Fig 7:**
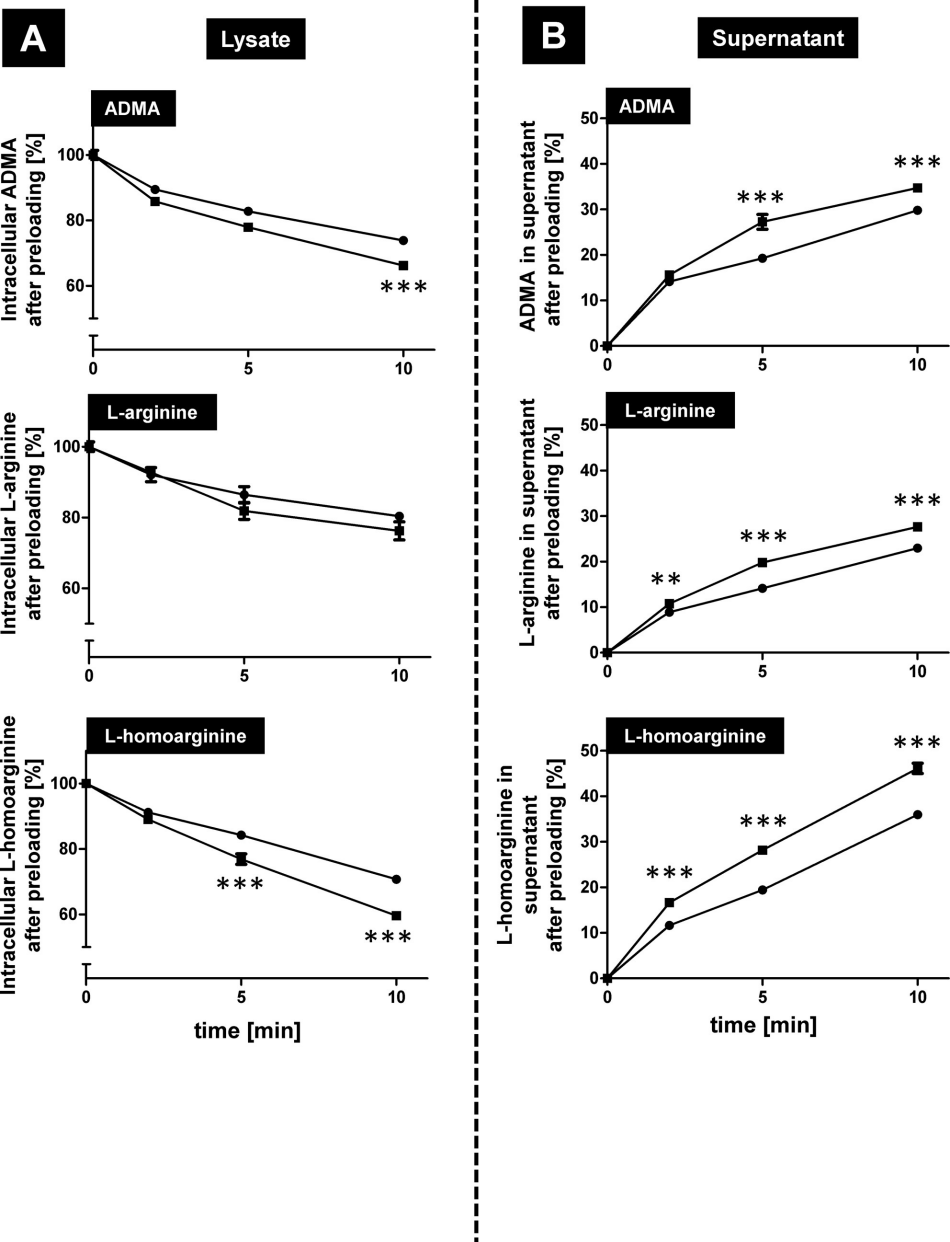
Investigation of OATP4C1-mediated export of ADMA, L-arginine and L-homoarginine. After preloading the cells with the respective compound (300 μM), the substrate concentration was measured in the cell lysate (A) and in the supernatant (B) of HEK-OATP4C1 cells (squares) and HEK-VC cells (circles). The radioactivity in the cells initially after preloading was set to 100% and the values are given related to this concentration. Data are expressed as means ± SEM. Experiments were performed on two separate days with at least n = 3 (n ≥ 6). **P < 0.01; ***P < 0.001.

Using the published OATP4C1 substrate digoxin for uptake experiments, we detected a significantly reduced intracellular accumulation of digoxin in HEK-OATP4C1 cells in comparison to the uptake into HEK-VC cells under standardized uptake conditions (P < 0.001; S1A Fig). This difference was abolished by the (non-selective) inhibitor cyclosporine A (S1B Fig). These findings point to an OATP4C1-mediated efflux of digoxin. To study possible OATP4C1-mediated efflux with respect to the arginine derivatives, preloading experiments were performed. After preloading the cells with 300 μM of the respective substrate, the remaining intracellular radioactivity in the lysate (Fig 7A) and the appearance of radioactivity in the supernatant (Fig 7B) was measured immediately after preloading and after 2, 5 and 10 minutes. For ADMA and L-homoarginine these preloading experiments demonstrated a highly significant reduction in radioactivity in the lysate of HEK-OATP4C1 cells compared to HEK-VC cells, accompanied by a significant increase in radioactivity in the respective supernatant (Fig 7A and 7B). For L-arginine this significant increase in the supernatant could also be detected (Fig 7B), but only a slight and not significant intracellular decrease was measured (Fig 7A).

**Fig 8:**
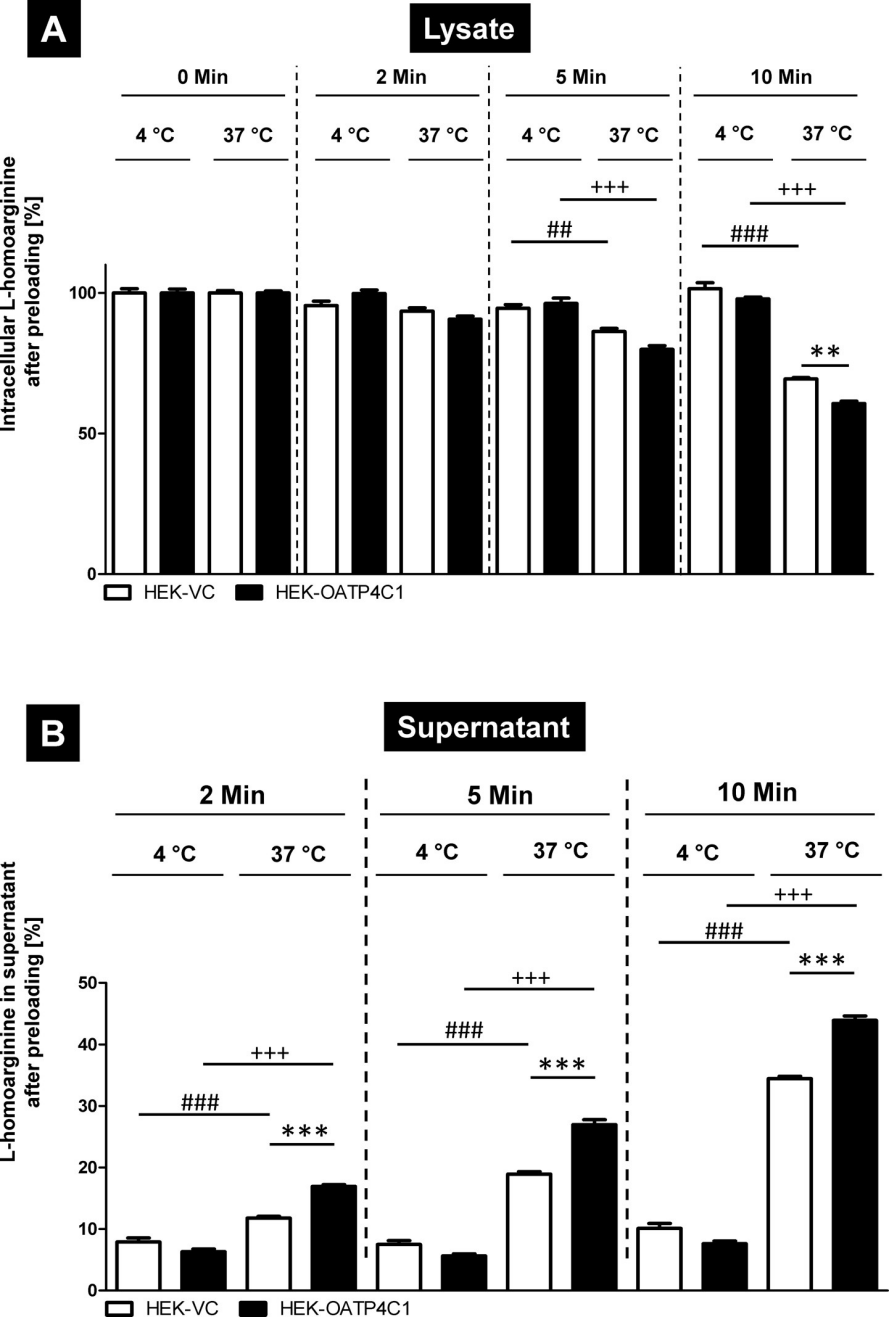
Temperature dependency of OATP4C1-mediated L-homoarginine efflux after preloading the cells. After preloading the cells, the substrate concentration was measured in the cell lysate (A) and in the supernatant (B) of HEK-OATP4C1 cells (black bars) and HEK-VC cells (white bars) with an incubation temperature of 37 ºC and 4 ºC. The radioactivity in the cells directly after preloading was set to 100% and the other values are given relative to this concentration. The studies were performed in two experiments with n = 2 (n = 4). ^##^P < 0.01 37 ºC HEK-VC versus 4 ºC HEK-VC; ^###^P < 0.001 37 ºC HEK-VC versus 4 ºC HEK-VC; **P < 0.01 37 ºC HEK-OATP4C1 versus 37 ºC HEK-VC; ***P < 0.001 37 ºC HEK-OATP4C1 versus 37 ºC HEK-VC; ^+++^P < 0.001 37 ºC HEK-OATP4C1 versus 4 ºC HEK-OATP4C1; one-way ANOVA with Bonferroni`s multiple comparison test.

To test, if this export from HEK-OATP4C1 and HEK-VC cells was a transporter-mediated process, experiments were repeated with L-homoarginine as substrate at 4 ºC and 37 ºC. As expected, at 37 ºC a significant increase in radioactivity in the supernatant of the HEK-OATP4C1 cells could be detected after 2, 5 and 10 minutes compared to HEK-VC cells (Fig 8B). At the same temperature, the amount of radioactivity was reduced in the lysate (Fig 8A) of the HEK-OATP4C1 cells (significant reduction after 10 minutes). At 4 ºC, no significant differences between HEK-OATP4C1 and HEK-VC cells could be detected at the examined time points. However, highly significant differences could be measured in the supernatant after 2, 5 and 10 minutes (Fig 8B) and in the lysate after 5 and 10 minutes (Fig 8A) comparing HEK-OATP4C1 cells incubated at 4 ºC and 37 ºC demonstrating that this L-homoarginine efflux is transporter-mediated.

## Discussion

Toyohara and coworkers [13] showed in a landmark study that the transport protein OATP4C1 could be involved in the renal secretion of selected uremic toxins. It was therefore the aim of our study to investigate the role of OATP4C1 for the transport of the uremic toxin ADMA and the structurally related substances L-homoarginine and L-arginine. We established stably-transfected HEK cells recombinantly overexpressing the human OATP4C1 protein 100% identical to the protein encoded by the reference sequence (NM_180991.4). Since overexpression of human OATP4C1 in transgenic rats decreased plasma concentrations of the uremic toxin ADMA [13], we first tested OATP4C1-mediated uptake of the uremic toxin ADMA, of L-arginine and of the cardioprotective biomarker L-homoarginine. All three substances could be identified as substrates of human OATP4C1 (Figs 2 and 4). Furthermore, ADMA and the closely related uremic toxin SDMA inhibited OATP4C1-mediated L-homoarginine uptake in a dose-dependent manner (Fig 5) with IC_50_ values of 116 μM and 54 μM, respectively, whereas other known uremic toxins had no effect on OATP4C1-mediated L-homoarginine uptake (Fig 6). L-homoarginine can also be exported by OATP4C1 out of cells as demonstrated by preloading experiments (Figs 7 and 8).

As already indicated above, several studies have demonstrated that high ADMA plasma concentrations are associated with all-cause mortality and incident cardiovascular events [10], whereas on the other hand low L-homoarginine plasma concentrations have been identified as an independent risk marker for renal, cerebrovascular and cardiovascular diseases [2,3]. This raises questions regarding the role of the renal transport protein OATP4C1 for the transport of both substances across the basolateral membrane of proximal tubule cells. As identified by Genome Wide Association Studies (GWAS), the major enzyme for endogenous L-homoarginine synthesis seems to be the L-arginine:glycine amidinotransferase (AGAT; also known as GAMT = Guanidinoacetate N-methyltransferase [5,6]), which is highly expressed in brain, liver and kidney [18]. Therefore, based on our data, intracellularly synthesized L-homoarginine can be exported out of proximal tubule cells by OATP4C1 whereas extracellularly located ADMA can be taken up from blood into proximal tubule cells by OATP4C1. Intracellularly, ADMA is metabolized by the enzyme dimethylarginine dimethylaminohydrolase 1 [DDAH1 [19]], which is expressed in human proximal tubule cells and therefore preventing ADMA from being re-exported into the systemic circulation by OATP4C1. This hypothesis is supported by the fact that several studies have observed a moderate decline in L-homoarginine plasma concentrations with advancing age [20], which can be, at least in part, be explained by reduced kidney function [2]. Normal L-homoarginine serum concentrations are around 2.6 μM [21], thus, 3–4 times higher compared to ADMA concentrations [21]. During renal impairment, ADMA concentrations can increase up to 8 μM [22], possibly inhibiting OATP4C1-mediated L-homoarginine secretion at high concentrations, as demonstrated *in vitro* for the inhibition of digoxin efflux by extracellularly added cyclosporine A and L-homoarginine (S1B and S2A Figs).

Furthermore, the findings presented in this manuscript are in line with the observations in a transgenic rat model overexpressing human OATP4C1 [13], which demonstrated that the overexpression of human OATP4C1 decreased plasma concentrations of ADMA accompanied by reduced hypertension, cardiomegaly and inflammation. OATP4C1-mediated uptake of L-homoarginine could be inhibited by the uremic toxins ADMA and SDMA, but not by other known uremic toxins, suggesting that more uptake transporters are involved in the renal secretion of this diverse group of endogenously synthesized metabolites [23]. Candidates for these transporters include members of the SLC22 family, especially OAT1 (gene symbol *SLC22A6*) and OAT3 (*SLC22A8*). Both transporters are expressed in renal proximal tubule cells and are capable of transporting several uremic toxins such as indoxyl sulfate, hippurate and indolacetate [24]. So far, ADMA has been identified as substrate for CAT1 and CAT2 (SLC7 family), for the SLC22 family member OCT2 and the SLC47 member MATE1 [14], whereas L-homoarginine could be characterized as substrate for the SLC7 family members CAT1, CAT2A and CAT2B [25]. Furthermore, other transporters of the SLC superfamily may be involved in the transport of arginine and its derivatives or of uremic toxins into proximal tubule cells.

During characterization of the stably-transfected cell lines we observed that previously published substrates of OATP4C1 [e.g. digoxin [26]] could not be confirmed as uptake substrates with our cell systems. In contrast, under our standardized uptake conditions adapted from studies investigating other OATP family members [14,17], we detected a significantly reduced accumulation of digoxin in HEK-OATP4C1 cells compared with vector-transfected HEK-VC cells (S1A Fig) suggesting an OATP4C1-mediated export of digoxin. Digoxin has a relatively high passive diffusion [1.25 μl/min^-1^ x mg protein^-1^ [27]] and the OATP4C1-mediated export out of HEK-OATP4C1 cells after digoxin entering the cells by passive diffusion may result in the reduced intracellular accumulation compared to HEK-VC cells. Interestingly, this transport can be inhibited by extracellularly added cyclosporine A and L-homoarginine (S1B and S2A Figs), whereas the uptake of L-homoarginine was also reduced when digoxin is added extracellularly (S2B Fig), possibly by trans-inhibiting OATP4C1-mediated L-homoarginine uptake. These results are in line with a study by He and coworkers [28], in which the authors investigated the interaction between digoxin and bupropion and found that bupropion increased the renal clearance of digoxin by 80%. The authors suggest that this was due to the inhibition of digoxin transport during reabsorption, possibly by inhibiting the OATP4C1-mediated transport back into blood.

Taken together, we established stably-transfected HEK cells recombinantly overexpressing the human renal transport protein OATP4C1 and characterized the uremic toxin ADMA as well as structurally related L-arginine and the protective factor L-homoarginine as substrates. Furthermore, we could demonstrate that this transporter can act bidirectionally suggesting a dual protective role of this transport protein. While L-homoarginine could be exported by OATP4C1 after being synthesized in proximal tubule cells, the OATP4C1-mediated uptake of ADMA might be a crucial step before intracellular ADMA degradation and subsequent renal excretion.

## Supporting information

S1 Fig Uptake of digoxin (5 μM) into HEK-OATP4C1 cells (HEK-OATP4C1) and HEK-VC cells (HEK-VC). (A) Uptake without added cyclosporine A (CsA). (B) Uptake with extracellularly added cyclosporine A (10 μM). Data are expressed as means ± SEM. Experiments were conducted on two separate days with n = 3 each (n = 6). ***P < 0.001.
(TIF)

S2 Fig Interaction of Digoxin and L-homoarginine regarding OATP4C1-mediated transport. (A) Net uptake of digoxin (5 μM) with (+ 20 μM L-homoarginine) and without (without) extracellularly added L-homoarginine (20 μM) demonstrating that L-homoarginine inhibited OATP4C1-mediated digoxin efflux. (B) Net uptake of L-homoarginine (20 μM) with (+ 5 μM Digoxin) and without (without) extracellularly added digoxin (5 μM). Data are expressed as means ± SEM. Experiments were conducted on two separate days with n = 3 each (n = 6) *P < 0.05; ***P < 0.001.
(TIF)
